# Teacher–Student Interaction and Chinese Students’ Mathematics Learning Outcomes: The Mediation of Mathematics Achievement Emotions

**DOI:** 10.3390/ijerph17134742

**Published:** 2020-07-01

**Authors:** Wei Lin, Hongbiao Yin, Jiwei Han, Jiying Han

**Affiliations:** 1 School of Educational Science, South China Normal University, Guangzhou 510631, China; linweiszjky@foxmail.com; 2 Shenzhen Institute of Educational Science Research, Shenzhen 518035, China; 3 Department of Curriculum & Instruction, Faculty of Education, Chinese University of Hong Kong, Hong Kong, China; yinhb@cuhk.edu.hk; 4 School of Mathematics and Statistics, Northeast Normal University, Changchun 130024, China; 5 School of Foreign Languages and Literature, Shandong University, Jinan 250100, China; jyhan@sdu.edu.cn

**Keywords:** teacher–student interaction, classroom environment, mathematics achievement emotions, mathematics learning outcomes, China

## Abstract

This study aimed to investigate secondary students’ mathematics achievement emotions and their mediating effects on the relationships between classroom environmental characteristics, namely, teacher–student interactional styles (i.e., teacher leadership and student freedom styles), and students’ mathematics learning outcomes in mainland China. A sample of 1423 Grade 7 to 9 junior secondary students responded to a questionnaire that comprised three sets of scales for assessing students’ perceived teacher–student interactional styles, mathematics achievement emotions, and cognitive and affective learning outcomes. The results indicated that students’ mathematics learning outcomes were positively associated with both teacher leadership and student freedom styles. Moreover, students’ mathematics achievement emotions mediated the relationships between these two interactional styles and their mathematics learning outcomes. These results highlight the importance of mathematics achievement emotions in student learning, and provide implications for the improvement of mathematics classroom environments.

## 1. Introduction

Traditionally, Chinese students who receive expository and teacher-centered instruction are considered to be rote learners, which is deemed as a less favorable educational environment by Western educators [1]. Although the educational reforms in China suggested that traditional lecturing should give way to student-centered approaches [2], the time-consuming nature of these approaches along with their unpredictability impede teachers’ decisions to adopt these methods [3]. Teachers in China are inclined to adopt a more teacher-centered manner to secure complete and effective teaching [4]. However, there is an apparent contradiction between the teacher-dominated classroom environment and the outstanding mathematics performance of students in international assessments such as the Programme for International Student Assessment and the Trends in International Mathematics and Science Study. Biggs even argued that both “teacher-centered” and “student-centered” approaches may exist simultaneously in Chinese mathematics classrooms “Teacher-centered approaches” refer to the teaching methods where teachers dominate and actively engage in teaching and classroom interactions in order to effectively transmit knowledge to students, while “student-centered approaches” put the responsibility of learning on the students and advocate that the students have the autonomy to choose the content of study and initiate classroom interactions [5]. Debunking the myth of Chinese learners and mathematics teachers’ interactional styles in classrooms has thus become an interesting topic [6,7].

Mathematics achievement emotions have also been widely recognized by researchers in recent years [8,9,10]. Sufficient evidence has demonstrated that achievement emotions play an important role in students’ learning and academic attainment [10,11]. Research on achievement emotions related to mathematics learning has shown significant associations between students’ emotional experiences, classroom situations, and their mathematics achievement [9]. Many studies have revealed that positive mathematics achievement emotions (e.g., enjoyment and pride) are positively related to students’ mathematics learning [12,13,14,15,16,17], while negative mathematics achievement emotions (e.g., anxiety and anger) negatively predict students’ mathematics achievement [12,18]. Moreover, it has been suggested that achievement emotions should be examined from the between-person perspective rather than as a within-person functional relationship [10].

Teacher–student interactions embodied in classroom environments have been confirmed to be associated with students’ motivation and emotions [19,20,21]. Therefore, the purpose of this research is to investigate how two selected teachers’ interactional styles (i.e., teacher leadership and student freedom) affect students’ mathematics achievement emotions and learning outcomes in the context of mainland China. Considering the characteristics of classroom environment in terms of teacher–student interactions in China, we attempt to explore how “teacher-centered” and “student-centered” interactions affect students’ mathematics achievement emotions and their learning outcomes.

## 2. Theoretical Perspectives

### 2.1. Mathematics Achievement Emotions

Achievement emotions are defined as emotions that are tied directly to achievement activities or achievement outcomes [10,22]. Control-value theory presented by Pekrun served as a theoretical framework of the present study [22]. This comprehensive theoretical approach integrates the attributional theories of achievement emotions, the expectancy value approaches to emotions, and perceived control theories, etc. Theoretically, “control” refers to individuals’ intervention on the mediators, which affects the related outcomes they perceive, in order to change their own behaviors and outcomes. While “value” is the importance of activities and outcomes that individuals realize, control-value theory describes two groups of appraisals related to achievement emotions [22]. First, individuals’ cognition depends on their control of achievement-related activities and outcomes. For example, if a learner continues to study hard, he or she will have more confidence in controlling the successful result. Individuals’ achievement-related competence beliefs, expectancies, and attributions are involved in the control-related appraisals. Second, individual cognition depends on their judgments of the achievement-related behaviors and the value of the outcomes. For example, if an individual recognizes the importance of success, he or she will commit more to that. In general, perceived controllability and positive value judgement of activities are inclined to arouse positive activity emotions.

In terms of the assessment of achievement emotions, Pekrun proposed the “valence × activation” pattern. Four positive emotions (joy, hope, pride, and relief) and five negative emotions (anger, anxiety, disappointment, shame, and boredom) were found to be common in students’ learning experiences [22]. Further, these emotions were classified by whether or not they were activated [23]. According to the criteria, the assessment of achievement emotions can be concluded by two dimensions: valence—positive emotions and negative emotions, and activation—activated emotions and inactive emotions. On the basis of these two dimensions, achievement emotions are divided into four categories: positive activated emotions (joy, hope, pride), positive inactivated emotions (relief), negative activated emotions (anger, anxiety, shame), and negative inactivated emotions (disappointment, boredom). In the present study, following Pekrun’s approach, we mainly focused on the activated mathematics achievement emotions (i.e., joy, pride, anger, anxiety, and shame) [22].

A number of studies have pointed out the importance of students’ achievement emotions in student learning [11,24]. Pekrun conducted research involving final-year college students, controlling for the related factors of students’ gender, intelligence, and family socio-economic status [24]. The results demonstrated that positive emotions (enjoyment, pride) positively predicted, while negative emotions (anger, anxiety, shame, boredom, hopelessness) negatively predicted students’ mathematics scores. These findings robustly confirmed that children’s and adolescents’ emotions are significantly linked to their academic achievement, highlighting the importance of achievement emotions in students’ school tracks. Other studies have focused on the relationship between students’ emotional experiences and learning environments. For example, previous studies have revealed that students’ emotions in mathematics were additionally linked to classes’ aggregate environment perceptions [19]. Peixoto et al. found that students’ emotional experiences were significantly associated with their competence and value appraisals in different learning contexts [9].

### 2.2. Teacher Interpersonal Behaviors

Teacher–student interaction constitutes an important dimension of the psychosocial environment in classrooms and has been reported to have significant effects on students’ motivation, engagement, and academic achievement [25,26,27]. Teacher interpersonal behavior, proposed by Wubbels and Brekelmans, denotes “a system approach to communication which was adapted to the educational setting to describe teacher behavior within the classroom setting” [28] (p. 608). Researchers have conceptualized different patterns of teacher–student interaction in terms of teacher interpersonal behavior [29]. The interpersonal theory embedded in a system approach to communication is the underlying theoretical foundation of the present study.

On the basis of the early model developed by Leary to allow the graphic representation of interpersonal behaviors, Wubbels et al. constructed a model for interpersonal teacher behavior that depicts teacher–student interaction along two dimensions, namely, influence and proximity [28,30]. Specifically, “influence” refers to the degree to which teachers control communication in the classroom, and “proximity” refers to the degree to which teachers cooperate with students. The model used an influence dimension (dominance, D; submission, S) and a proximity dimension (cooperation, C; opposition, O) to measure the degree of dominance or control and the degree of affinity or cooperation over the classroom communication process, respectively. Furthermore, Wubbels et al. detailed the model into eight sectors, namely, leadership (DC), helping/friendly (CD), understanding (CS), student responsibility/freedom (SC), uncertain (SO), dissatisfied (OS), admonishing (OD), and strict (DO). Each sector describes different facets of teacher interactional behaviors in classrooms [31].

Considering the characteristics of teacher–student interaction in China, the present study selected two types of teacher interactional behaviors. Both reflect some typical teacher–student relationships in Chinese classrooms. These are (1) teacher leadership (DC) interaction, which means that teachers lead or organize everything that happens in the classroom. They may give orders or frequently set tasks for students. They pay more attention to procedures and structure in teaching or in other classroom situations. They are good at explaining thoroughly to students, as well as holding all students’ attention in class. (2) Student responsibility/freedom (SC) interaction, which means teachers will give students more opportunities and autonomy to carry out independent work. Teachers are patient in waiting for students to form their self-regulation. They encourage students to take responsibility for their own studies and give them more opportunities to initiate and sustain the learning tasks.

### 2.3. The Characteristics of Teacher–Student Interaction in Chinese Classrooms

In China, the development of teacher–student relationships is affected by Confucianism. The traditional virtues of “respecting teachers and valuing the way” (*Xue Ji*) has always been deemed to be important since ancient times in China. This highlights the importance of respecting teachers in China. Influenced by this cultural and educational tradition, Chinese students have strong faith in the virtue of “wherever respect exists, teachers exist” (*Xue Ji*) and traditionally respect teachers, leading to the long-standing teacher-centeredness as the dominant pattern in Chinese classrooms.

However, the teacher-centered classroom environments have been challenged and have been seen as an inefficient practice in the past two decades. Instead, student-centeredness is claimed to be one of the most promising directions in current educational and policy discourses [32]. Influenced by the global educational reform policies, curriculum reforms advocating student-centeredness have also emerged in China [33]. The new round of curriculum reforms since 2001 has clearly proposed a shift in teacher–student relationships from teacher-centeredness to student-centeredness. Teachers are required to respect students’ different learning needs, as well as to create an equal relationship between teachers and students. Student-centered approaches have been shown to have some impact on teaching effectiveness, such as obtaining higher grades and developing enhanced perceptions of students’ abilities [34]. However, “student-centered” interactions are inclined to be time-consuming and unpredictable, which makes Chinese teachers uncertain about the adoption of student-centered approaches. In contrast, Wang proposed that the traditional teacher-centered interactions are helpful for saving time, for providing students with chances to successfully “get it”, and for enabling teachers to complete tasks [4]. Teachers are more comfortable with the teacher-centered pedagogy in order to prepare well for standardized tests and high-stakes examinations [35]. Consequently, Chinese teachers often face the dilemma of adopting teacher-centered versus student-centered approaches in the classroom.

At the same time, there are two paradoxes in understanding Chinese teacher–student interactions. First, the excellent mathematics performance of Asian students in the past decade has shaken Western scholars’ firm confidence in “student-centeredness” conceptions. Why can Chinese learners, who are labeled as “traditional, rigid, passive, and even uninquisitive”, outperform others in mathematics? This question has repeatedly puzzled researchers and practitioners. Second, should teacher-centeredness be replaced by student-centeredness as advocated by the recent Chinese curriculum reforms? This is still “an unproved proposition” [36]. With the development of curriculum reforms in China, there are more diverse voices discussing teacher–student relationships. Some researchers believe that the core of teaching is the student, while teachers are only the facilitators of student learning [37]. Meanwhile, some empirical research has presented that teacher-centeredness is not opposite to student-centeredness, as both can guide students to actively participate in the classroom [38]. Kain even proposed collapsing the binary of “teacher-centeredness” and “student-centeredness” [34]. Hence, the present study attempts to explore which one is more effective in terms of improving students’ mathematics learning in the context of classrooms in China.

The way students perceive their teacher’s interpersonal behavior has been reported to be related to the students’ learning motivation, attitude, pleasure, relevance, confidence, and effort in specific subjects [31,39]. Numerous studies have shown that the proximity and influence dimensions of teachers’ interpersonal behavior are positively associated with students’ enjoyment and interest in mathematics, science, and English learning [40,41]. Previous studies have also shown that students’ mathematics achievement emotions are affected by their achievement goals, personality antecedents, and socio-cultural environments, etc. [42]. The research about environmental variables and emotional experiences has further revealed that the class’ aggregate environment perceptions of teacher behaviors are associated with students’ emotions in mathematics. Moreover, children’s and adolescents’ emotions are linked to their academic achievement and performance [24]. Therefore, the associations between teachers’ interpersonal behaviors and students’ achievement emotions as well as their learning performances are worthy of investigation in the context of China.

In short, this study aims at addressing three questions: (1) What are the effects of two types of teacher–student interaction, i.e., teacher leadership and student freedom, on students’ mathematics achievement emotions? (2) What are the effects of these two types of teacher–student interactions on students’ mathematics learning outcomes? (3) Is there any significant mediation effect of students’ mathematics achievement emotions on the relationships between teachers’ interpersonal behaviors and students’ mathematics learning outcomes?

## 3. Methods

### 3.1. Participants

Consistent with research ethics review procedures, we carried out this study in accordance with the recommendations of the Survey and Behavioral Research Ethics Committee at the institute where the authors work.

Convenience sampling was used in this study. A questionnaire survey was conducted for data collection from January to April 2017 in Shenzhen. A total of 2000 copies of the questionnaire were distributed to 22 classes from 11 schools (2 classes per school), and finally 1423 completed questionnaires were returned, giving a response rate of 71.5%.

The sample consisted of 796 males (55.9%) and 627 females (44.1%); 606 Grade 7 students (42.6%), 600 Grade 8 students (42.1%), and 217 Grade 9 students (15.3%). With regards to their school background, 992 (69.7%) of them came from public schools and 431 (30.3%) came from private schools; 399 (28%) came from high-achieving key schools and 1024 (72%) were from low-achieving ordinary schools.

### 3.2. Measures

Apart from a uniform mathematics test, a questionnaire comprising three scales including the Questionnaire on Teacher Interaction (QTI), Achievement Emotions Questionnaire-Mathematics (AEQ-M), and affective mathematics learning outcomes extracted from the PISA 2012 mathematics test were used in this study. The Chinese versions of these scales were administered in the present study. To ensure the quality of the translation of QTI, two authors who were fluent in both Chinese and English independently conducted translation and back translation of selected QTI subscales. The Chinese version of AEQ-M validated by Pekrun and PISA 2012 (Chinese version) were adopted directly in this study [24]. The participants were asked to rate each item on a 4-point Likert scale ranging from *strongly disagree* to *strongly agree*.

It is noted that teacher–student interaction was assessed from the students’ perceptions rather than teachers’. In learning environment research that focuses on the psychosocial climates in the classroom, researchers often found that there are some differences between students’ and teachers’ reported classroom environments. However, compared with teachers’ perceptions, students’ perceptions of the classroom environment have been given more importance in eliciting their psychological and behavioral reactions in learning [43,44]. Therefore, we chose to assess teacher–student interaction in the classroom from the perspective of students in this study. Meanwhile, to avoid the over-reliance on students’ self-report, we specifically adopted a uniform mathematics test.

#### 3.2.1. Teacher Leadership and Student Freedom

Two selected QTI subscales developed by Fisher, Fraser, and Wubbels to assess teacher–student interactions were translated into Chinese [45]. The original QTI contains 8 dimensions assessed by 48 items, with 6 for each dimension. This study used 12 items extracted from the QTI to assess the 2 styles of teacher–student interaction, namely, teacher leadership (e.g., “We all listen to this teacher”) and student freedom (e.g., “This teacher allows us to work on things that we like”). Each dimension comprises 6 items. The QTI scale selected in the present study is shown in Appendix A.

#### 3.2.2. Mathematics Achievement Emotions

The Chinese version of the 37-item AEQ-M developed by Frenzel et al. was used to assess students’ achievement emotions in mathematics learning [19]. This scale measured 5 achievement emotions: enjoyment (9 items; e.g., “I look forward to the mathematics class”), pride (6 items; e.g., “I think that I can be proud of my knowledge”), anger (8 items; e.g., “I get angry while studying math”), anxiety (6 items; e.g., “I feel nervous in math class”), and shame (8 items; e.g., “I feel ashamed for not being able to answer the math teacher’s question well”).

In the AEQ-M, the items are ordered in 3 blocks that assess students’ emotional experiences before, during, and after being in achievement situations (i.e., before, during, and after they conduct some mathematics learning tasks). In this study, considering the immediacy and intimate relationship between emotions and behaviors in student learning, we chose to assess students’ learning-related emotions during their mathematics learning in classrooms. In this context, achievement emotions may better play their role in triggering students’ learning performance. The AEQ-M scale used in the present study is also shown in Appendix B.

#### 3.2.3. Affective Mathematics Learning Outcomes

This study used 8 items extracted from the PISA 2012 mathematics test (Chinese version) to assess students’ affective mathematics learning outcomes, including students’ motivation for mathematics (e.g., “Making an effort in mathematics is worth it because it will help me in the work that I want to do later on”) and their mathematics self-concept (e.g., “I have always believed that mathematics is one of my best subjects”). Each dimension has 4 items. The affective mathematics learning outcomes scale selected from PISA 2012 in the present study is also shown in Appendix C.

Because the scales listed above are all self-report, Harman’s single-factor test was conducted to address the potential common method bias [46]. The one-factor solution of exploratory factor analysis only accounted for 24.83% of the variance, indicating that common method bias was not a big concern in this study.

#### 3.2.4. Mathematics Achievement Test

In this study, students’ mathematics achievement was assessed by a uniform examination designed by the local Education Bureau of Shenzhen. This examination was constructed with reference to the Chinese mathematics curriculum standards of mathematics ability in middle schools. The examination takes basic mathematics knowledge in each academic year as the main content, divided into the 4 areas of number and algebra, space and graphics, probability and statistics, and problem-solving. In terms of the patterns, this examination included different kinds of questions such as fill-in-the-blank, multiple choice, judgment, calculation, and problem-solving.

### 3.3. Statistical Analysis

This study used SPSS 22.0 (IBM Corp, Armonk, NY, USA) and AMOS 17.0 (IBM Corp, Armonk, NY, USA) to analyze the data. Structural equation modeling (SEM) was used to examine the relationships between the constructs of interest. The whole test process followed the two-step procedure. First, confirmatory factor analysis (CFA) was conducted to examine the construct validity. Then, the descriptive statistics and correlations were calculated by SPSS 22.0. Second, the method of structural equation modeling (SEM) and mediation analysis were conducted using AMOS 17.0. The model fit was assessed using the χ² statistics, and the comparative fit index (CFI), the Tucker–Lewis index (TLI), and the root-mean-square error of approximation (RMSEA) were the indices used to assess the goodness-of-fit. The acceptable and excellent model fit requires, respectively, the CFI and TLI values to be no less than 0.90 and 0.95, and the RMSEA value to be less than 0.06 and 0.08 [47]. Moreover, bootstrapping, one of the most powerful and sensible methods for detecting indirect effect [48], was employed to examine the mediated effects.

## 4. Results

### 4.1. Construct Validity and Reliability

To examine whether the data fit the original measurement model in this study, we conducted maximum-likelihood CFA using AMOS 17.0. The reliability and construct validity of both the AEQ-M and QTI subscales and affective mathematics learning outcomes questionnaire were examined.

For AEQ-M, the results showed that the five achievement emotions had acceptable reliability coefficients; the Cronbach’s α reliability coefficients for the five sub-scales, as presented in Table 1, were 0.88 (enjoyment), 0.81 (pride), 0.86 (anger), 0.73 (anxiety), and 0.80 (shame), indicating good internal consistencies for each sub-scale. The first-order factor structure showed a marginally acceptable model fit (χ² = 2352.52, df = 470, *p* < 0.001, CFI = 0.90, TLI = 0.89, RMSEA = 0.053). All items had factor loadings ranging from 0.40 to 0.80. However, some factors were highly correlated, ranging from 0.60 to 0.70, suggesting that a second-order factor structure could better explain achievement emotions. Therefore, a second-order factor structure consisting of positive (enjoyment and pride) and negative (anger, anxiety, and shame) achievement emotions was examined. The results showed that this second-order CFA reached an acceptable model fit (χ² = 2573.779, df = 367, *p* < 0.001, CFI = 0.90, TLI = 0.90, RMSEA = 0.065).

CFA was used to examine the construct validity of two QTI subscales. The initial CFA results indicated that item 8 (“This teacher makes us work hard”), item 10 (“We have to be quiet in this teacher’s class”), and item 12 (“This teacher’s tests are hard”) had low factor loadings (< 0.40). Hence, these items were deleted from the scale. The goodness-of-fit indices of the modified model revealed that these deletions substantially improved the model fit—for teacher leadership, the fit indices were χ² = 36.1, df = 6, *p* < 0.01, CFI = 0.97, TLI = 0.93, RMSEA = 0.059; and for student freedom, they were χ² = 6.8, df = 4, *p* < 0.01, CFI = 0.99, TLI = 0.98, RMSEA = 0.002. The Cronbach’s alpha values for the two dimensions were 0.71 (leadership) and 0.68 (freedom), respectively, suggesting acceptable internal consistency.

In terms of the affective mathematics learning outcomes, the goodness of fit indices were χ² = 675.10, df = 112, *p* < 0.01, CFI = 0.95, TLI = 0.95, RMSEA = 0.059, and the factor loadings of all items ranged from 0.67 to 0.88. The Cronbach’s alpha for the two sub-scales were 0.89 (mathematics motivation) and 0.88 (self-concept), respectively.

### 4.2. Descriptive Statistics and Correlations

The Cronbach’s alpha coefficient of each factor, the correlation matrix among factors, and descriptive statistics are shown in Table 1. Teachers’ leadership interactions scored higher (Mean = 4.04, SD = 0.58) than freedom interactions (Mean = 3.08, SD = 0.52). Among the five factors of AEQ, students’ positive emotions included enjoyment (Mean = 3.49, SD = 0.78) and pride (Mean = 3.49, SD = 0.78), which had the most positive scores, and students’ anger (Mean = 2.05, SD = 0.76), which scored the lowest. As for the affective mathematics learning outcomes questionnaire, students’ mathematics motivation (Mean = 3.06, SD = 0.58) scored higher than their self-concept (Mean = 2.43, SD = 0.76).

Table 1 also shows the correlation matrix for all of the factors. All variables observed in this study were moderately correlated with each other. Selected teachers’ interpersonal behaviors (teacher leadership and student freedom) were positively correlated to all students’ positive achievement emotions (enjoyment and pride) and their mathematic learning outcomes, but were negatively correlated with students’ negative achievement emotions (anger, anxiety, and shame).

### 4.3. SEM Analysis

**Figure 1:**
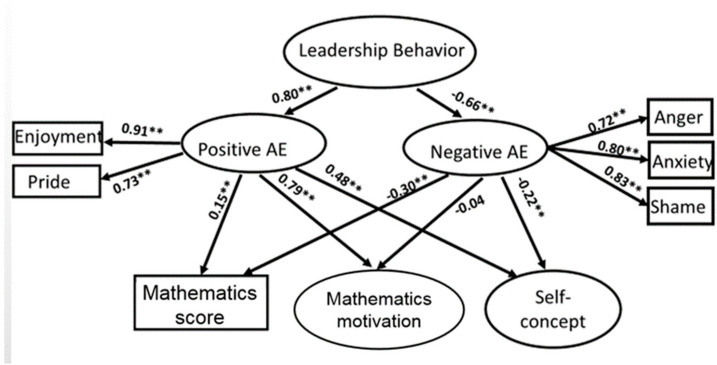
Impact of leadership interpersonal behavior on students’ mathematics performance through the mediation of achievement emotions (AE = mathematics achievement emotions). Note: ** *p* < 0.01.

**Figure 2:**
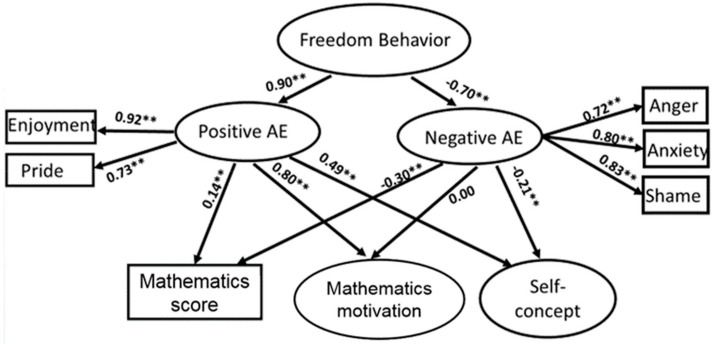
Impact of freedom interpersonal behavior on students’ mathematics performance through the mediation of achievement emotions (AE = mathematics achievement emotions). Note: ** *p* < 0.01.

This study followed the person–environment fit theory [49] to test the relationships between the environmental factors (i.e., teacher interactional behaviors), personal factors (i.e., mathematics achievement emotions), and performance (i.e., cognitive and affective learning outcomes). Two SEM models were constructed and tested to address the research questions. Both models reached an acceptable model fit. For the teacher leadership model (Figure 1), χ² = 1537.88, df = 240, *p* < 0.01, RMSEA = 0.065, CFI = 0.92, TLI = 0.90; for the student freedom model (Figure 2), χ² = 1210.74, df = 179, *p* < 0.01, RMSEA = 0.064, CFI = 0.93, TLI = 0.91.

The results indicated that teacher leadership and student freedom styles were positively associated with students’ positive achievement emotions (β_leadership_ = 0.80, *p* < 0.01; β_student freedom_ = 0.90, *p* < 0.01) consisting of enjoyment and pride, and were negatively associated with students’ negative achievement emotions (β_leadership_ = −0.66, *p* < 0.01; β_student freedom_ = −0.70, *p* < 0.01) including anger, anxiety, and shame. The positive achievement emotions were all positively associated with students’ mathematics achievement (β_leadership_ = 0.15, *p* < 0.01; β_student freedom_ = 0.14, *p* < 0.01) and affective outcomes including motivation (β_leadership_ = 0.79, *p* < 0.01; β_student freedom_ = 0.80, *p* < 0.01) and self-concept (β_leadership_ = 0.48, *p* < 0.01; β_student freedom_ = 0.49, *p* < 0.01), while the negative achievement emotions were only negatively associated with students’ mathematics achievements (β_leadership_ = −0.30, *p* < 0.01; β_student freedom_ = −0.30, *p* < 0.01) and their self-concept (β_leadership_ = −0.22, *p* < 0.01; β_student freedom_ = −0.21, *p* < 0.01).

### 4.4. Mediation Analysis

The mediation analysis was conducted using 5000 bootstrapping samples. The results are presented in Table 2. In this study, the point estimate of the indirect effect (ab) was adopted to assess the effect size of the mediator. According to Hayes, the indirect effect is significant when zero is not between the lower and upper bound in the 95% confidence interval [48]. The bootstrapping results presented in Table 2 indicated that the mediation effects of teacher leadership and student freedom on students’ mathematics learning outcomes (i.e., test score, motivation, and self-concept) through students’ achievement emotions were significant.

## 5. Discussion

### 5.1. Characteristics of Chinese Teachers’ Interpersonal Behaviors and Students’ Achievement Emotions

As previous studies have revealed, teacher–student relationships in China have been debated for many years [37,38]. On one hand, results of the current study supported the studies conducted in Chinese contexts, emphasizing the importance of teacher leadership to Chinese learners. Lee and Yin presented that Chinese students’ development could be steadily improved by teachers’ support and help, even though teachers participated more than students in classroom activities [50]. Mok et al. also demonstrated that high teacher control in classroom teaching did not necessarily lead to negative learning outcomes for Chinese students [51]. On the other hand, student freedom interactions scored lower than teacher leadership in the present study. This may reveal some difficulties in creating a classroom environment characterized by student freedom [6]. As Wang pointed out, although the curriculum reforms in China emphasized student-centeredness, the drawbacks of being time-consuming, being unpredictable for the teacher, and the uncertainty about completing the required tasks impede the implementation of student-centeredness in classrooms [3,4].

The characteristics of Chinese students’ achievement emotions were also highlighted in the present study. Among the five dimensions, the students reported the highest score for the positive emotions including enjoyment and pride, indicating deep affection and great enthusiasm for mathematics learning. Meanwhile, they reported the highest score in negative achievement emotions for mathematics anxiety, indicating the heavy pressure of high-stakes examinations as well as the difficulty of mathematics tasks in China.

### 5.2. The Positive Effects of Both Teacher-Centered and Student-Centered Classroom Environments

The study found that both teacher leadership and student freedom interactional styles had similar positive effects on students’ mathematics learning outcomes, supporting the effectiveness of both teacher-centered and student-centered classroom environments. Researchers have proposed that “teacher-centeredness” and “student-centeredness” are not polarized in the cultural contexts of China [38]. Teachers in mainland China will take corresponding actions under different circumstances in order to make use of “teacher-centeredness” and “student-centeredness” at the same time [52]. Similarly, we found that students’ mathematics learning outcomes were significantly improved by both teacher leadership and student freedom styles.

Actually, teacher-centeredness in China is far from the stereotypical understanding of tyrannical teachers, pure knowledge transmission, and so on. Through the professional guidance of teachers, students are more able to achieve deep learning. Lee and Yin suggested that “activating teaching” is a more accurate way to present the Chinese teaching style than teacher-centeredness [50]. Moreover, the Confucian teaching philosophy also reminds us that “teachers should not enlighten students until they have really tried hard and even failed to understand; teachers should not instruct them until they have something to say and can make themselves understood” (*The Analects*). It not only stresses the importance of students’ learning initiative, but also emphasizes the requirement for teachers to take on a guiding role.

In short, the results of the present study indicated that both teacher-centered and student-centered classroom environments may improve the effectiveness of students’ mathematics learning. Therefore, the teacher–student interaction in Chinese mathematics classrooms should combine the teacher’s guiding role and students’ initiative, rather than taking them as polarized opposites.

### 5.3. The Mediation Effects of Students’ Achievement Emotions

The results of mediation analysis revealed that both teacher leadership interactions and student freedom interactions had significant influences on students’ mathematics scores, motivations, and self-concepts via the mediation of students’ mathematics achievement emotions. These findings lend support to the rising argument that achievement emotions play important roles in students’ mathematics learning [8,9,16,17,19].

The findings of the present study could be explained by the following three possible reasons. Firstly, students’ cognitive resource allocation would be affected by individuals’ attention to achievement emotions [53]. Students may increase or decrease their focus on the specific task when they have different achievement emotions. Moreover, students’ learning strategies could be affected by their achievement emotions. Flexible and creative learning strategies are easier to be adopted when students have active and positive emotions, while simple and repetitive learning strategies are inclined to be used by students with activated and negative emotions (such as anger), and superficial strategies are prone to be used by students with inactivated and negative emotions (such as boredom) [54]. Consequently, the achievement emotion aroused by teachers’ interactional behaviors could impact students’ mathematics test scores. Secondly, students’ learning motivation could be affected by their achievement emotions [53]. The result of the present study revealed that students’ motivation would be enhanced by their positive emotions, while their interest in learning would be destroyed or weakened by their negative emotions. Thirdly, students’ self-regulation, which is helpful for forming self-concept, could be promoted by their positive achievement emotions, while negative emotions make students rely on external supervision, which is harmful for their self-concept construction [54].

The result of mediation analysis also revealed that the effect size of students’ mathematics achievement emotion as a mediator was larger for the effect of student freedom interactions than for teacher leadership interactions with regard tto the relationship with students’ mathematics learning outcomes. This indicates that for students who achieve better mathematics learning outcomes, the freedom interactions taken by teachers that provide students with stronger positive achievement emotions while impeding negative achievement emotions at the same time have a direct effect. Previous studies have demonstrated that students’ mathematics learning would be affected by their achievement emotions through different emotional intensity (important levels) and different directions (positive/negative) [55,56]. Pekrun pointed out that the strongest positive achievement emotion comes from the experience of the epiphany “ah!” when they solve a problem successfully, which fills them with satisfaction and pleasure [24]. On the other hand, the most intense and frequent negative achievement emotion comes from students encountering difficulties. Both of these two kinds of emotional experience come from students’ own learning settings. Therefore, student freedom interactions have the strongest power to inspire their positive achievement emotions in mathematics learning, which will enhance their leaning outcomes. This indicates that teachers should pay more attention to providing students with freedom for interactions, even in the context of China.

### 5.4. Limitations

Three limitations of the present study should be noted as they will also pave the way for future studies. Firstly, the present research is cross-sectional, focusing on the effects of classroom environment on students’ mathematics learning outcomes. We followed the person–environment fit theory to test the relationships between the variables of interest. However, other possible relationships between the variables of interest may exist. For example, students’ achievement emotions may in turn influence their perceptions of the classroom environment, whereas students’ motivation and self-concept may predict their mathematics achievement. Therefore, it is suggested that future researchers could consider using a longitudinal design to confirm the directionality of the paths. Secondly, all of the participants were from secondary schools in Shenzhen. Given that China is a vast country, the generalizability of the findings from the current study is limited. Therefore, it is suggested that future studies use some representative samples and include participants from different cities and regions. Third, due to convenience sampling and the focus of this study, we only examined the relationships between the variables of interest at the individual student level. However, data in school settings are nested in nature, and measurement invariance is also an interesting issue in statistics that often deserves a separate paper. This study did not take the measurement invariance and multilevel structure of the date into consideration during data analysis because of the limited numbers of schools and classes involved. Future research is highly suggested to improve the sampling method, enlarge the numbers of schools and classes, and deal with the multilevel and measurement invariance issues in data analysis.

## 6. Conclusions

The present study extends our understanding of the connections between teacher–student interaction, students’ mathematics achievement emotions, and their mathematics learning outcomes. The findings not only highlight some characteristics of Chinese teachers’ interactions and students’ achievement emotions, but also provide some evidence about how different types of teacher–student interaction affect students’ mathematics learning outcomes via mathematics achievement emotions. In brief, the results of the present study indicate that the factors influencing students’ mathematics learning outcomes are related to their mathematics achievement emotions and the teacher–student interaction in the classroom. 

These results bring some implications for improving mathematics teaching and classroom interaction. First, these results have some implications for the improvement of mathematics teaching in terms of how to effectively identify students’ achievement emotions. It is suggested that teachers pay attention to students’ achievement emotions, which should be seen as one of mathematics teachers’ basic skills. Teachers should be good at eliciting students’ positive emotional experiences. They also need to be skillful in altering students’ negative emotions such as anger, anxiety, and shame. For example, in classroom teaching, teachers are suggested to provide students with open and attractive questions at the beginning of class in order to arouse students’ curiosity and interest, ensuring their positive emotional states in learning. Moreover, teachers should arrange some clear and structured cognitive activities that are helpful to promote students’ sense of control. When students are solving a new problem, their feeling of confusion may impede their understanding of the problem. At that time, teachers are expected to intervene and help the students relieve their negative emotions.

Second, the results of this study indicate both teacher leadership and student freedom interactions in classrooms are effective in supporting students’ mathematics learning. However, teachers should pay attention to the processes and strategies for initiating and organizing the teaching activities. Specifically, teachers who incline to a “teacher leadership” interactional style tend to make everything well-organized. With the adequate preparation, these teachers are good at guiding students to think purposely and efficiently. They lead students to solve problems, think clearly, and check the answers step by step, which ensures the effectiveness of students’ mathematics learning. The “teacher leadership” interactional style also helps teachers to create an orderly classroom environment that facilitates students to think actively and solve problems efficiently. In contrast, teachers who incline to adopt a “student freedom” interactional style give students more autonomy and encourage students to take responsibility for their own learning. Since each student has different advantages and disadvantages, these teachers encourage students to face their unique problems, and help them take action and obtain better performance. Consequently, for teachers who incline to “student freedom” interactional style, they are more willing to try various pedagogies, including independent learning, peer learning, and problem-based learning, which may exert students’ autonomy and meet their different learning needs.

Third, good teachers use various approaches in their instruction [32]. Previous experimental studies have found that both teacher-centered and student-centered instructional approaches promote students’ understanding and engagement in science learning [57]. According to the results of this present study, Chinese mathematics teachers should be aware of the influence of their interactional behaviors in order to maximize students’ potential for learning. For example, it is suggested that teachers adopt leadership interactions when students head off in the wrong direction during problem-solving processes. At this time, teachers should actively intervene in order to get in the right direction. Meanwhile, teachers are advised to adopt free interactions when students are heading the right way towards the targets, so that students may have the freedom to solve the problems by themselves, as this can promote their higher-order thinking skills [32] and make them proud of their success [30]. Only when teachers choose the appropriate interactional behaviors in classroom settings can students’ autonomy and sense of responsibility in mathematics learning be effectively developed.

## Figures and Tables

**Table 1:** Descriptive statistics and correlation matrix.

Variable	1	2	3	4	5	6	7	8	9	10
1. Leadership	(0.71)									
2. Freedom	0.29 **	(0.68)								
3. Enjoyment	0.53 **	0.45 **	(0.88)							
4. Pride	0.43 **	0.36 **	0.71 **	(0.81)						
5. Anger	–0.47 **	–0.38 **	–0.62 **	–0.45 **	(0.86)					
6. Anxiety	–0.34 **	–0.30 **	–0.49 **	–0.32 **	0.59 **	(0.73)				
7. Shame	–0.37 **	–0.34 **	−0.45 **	−0.36 **	0.65 **	0.70 **	(0.80)			
8. Score	0.21 **	0.24 **	0.29 **	0.29 **	–0.26 **	–0.32 **	–0.33 **	-		
9. Motivation	0.46 **	0.34 **	0.67 **	0.51 **	–0.42 **	–0.36 **	–0.34 **	0.24 **	(0.89)	
10. Concept	0.23 **	0.20 **	0.51 **	0.43 **	–0.30 *	−0.46 **	–0.38 **	0.30 **	0.52 **	(0.88)
Mean	4.04	3.08	3.49	3.49	2.05	2.85	2.46	62.4	3.06	2.43
SD	0.58	0.52	0.78	0.78	0.76	0.77	0.74	1.08	0.58	0.76

Note: * *p* < 0.05; ** *p* < 0.01; Cronbach’s alpha coefficients are in parentheses along the diagonal.

**Table 2:** The results of mediation tests.

Dependent Variable	Independent Variable	Mediation Analysis					
		Point Estimate	Product of Coefficients		Bootstrapping		
					BC 95% CI		
		ab	SE	*p*	Lower	Upper	*R*²
Mathematics score	Leadership	0.29	0.082	0.03	0.23	0.35	0.17
	Freedom	0.30	0.086	0.00	0.24	0.35	0.18
Mathematics motivation	Leadership	0.67	0.055	0.04	0.61	0.72	0.69
	Freedom	0.70	0.074	0.00	0.61	0.78	0.67
Mathematics self-concept	Leadership	0.41	0.063	0.00	0.34	0.48	0.54
	Freedom	0.43	0.092	0.00	0.33	0.52	0.77

## Appendix A. The Selected QTI Subscales Used in the Study

### Appendix A.1. Leadership Behaviors

1. We all listen to this teacher.
2. This teacher doesn’t seem sure. (Reverse)
3. We learn a lot from this teacher.
4. This teacher is not sure of himself/herself. (Reverse)
5. This teacher gets our attention.
6. This teacher is shy. (Reverse)

### Appendix A.2. Freedom/Responsibility Behaviors

7. This teacher allows us to work on things that we like.
8. This teacher makes us work hard. (Reverse)
9. We can decide some things in this teacher’s class.
10. We have to be quiet in this teacher’s class. (Reverse)
11. This teacher gives us a lot of free time in class.
12. This teacher’s tests are hard. (Reverse)

## Appendix B. The AEQ-M Used in the Study

1. I look forward to the mathematics class.
2. I like mathematics class.
3. My enjoyment of this class makes me want to participate.
4. I get excited about going to class.
5. I enjoy taking the exam.
6. Hoping for success, I’m motivated to invest a lot of effort.
7. When I do my math homework, I am in a good mood.
8. I am happy that I understood the material.
9. I enjoy doing my math homework so much that I don’t want to stop.
10. I think that I can be proud of my knowledge.
11. I am proud of my answer to the math teacher’s question.
12. After the math exam, I felt proud of myself.
13. I’m proud of how well I mastered the exam.
14. Because I hope I can be proud of my math homework, so I am very motivated.
15. After finishing my math homework, I felt proud.
16. I get angry while studying math.
17. I am so angry that I want to leave math class.
18. I am angry by the difficulty of the math content.
19. When I take the math test, I want to tear it to pieces because I was so angry.
20. I am so angry after the math test.
21. I am annoyed at my math homework.
22. I get so angry to my math homework, I start feeling hot and flushed.
23. I get so angry I feel like throwing the math homework out of the window.
24. I feel nervous in math class.
25. I’m afraid that math is too complicated for me.
26. I feel nervous during the math exam.
27. When I take the math exam, I am worried that I wouldn’t do well.
28. I start sweating when I am worried I won’t finish my math homework.
29. I’m afraid I may not be able to master all the math at all.
30. I feel ashamed for not being able to answer the math teacher’s question well.
31. I feel myself blush when I answer questions in math class.
32. I feel hot on my face because I am embarrassed that I cannot answer the teacher’s question.
33. I feel so ashamed that I want to dig a hole.
34. I feel ashamed after the math exam.
35. When my teacher hand back the math test, I avoid looking my classmates in their eyes.
36. I feel ashamed when I realize that I lack basic math knowledge.
37. I don’t want to tell anyone when I don’t understand something in my math homework.

## Appendix C. The Measures of Affective Mathematics Learning Outcomes Used in the Study

### Appendix C.1. Motivation for Mathematics

1. I enjoy reading about mathematics.
2. Making an effort in mathematics is worth it because it will help me in the work that I want to do later on.
3. I look forward to my mathematics lessons.
4. I do mathematics because I enjoy it.
5. Learning mathematics is worthwhile for me because it will improve my career prospects.
6. I am interested in the things I learn in mathematics.
7. Mathematics is an important subject for me because I need it for what I want to study later on.
8. I will learn many things in mathematics that will help me get a job.

### Appendix C.2. Mathematics Self-Concept

1. I am just not good at mathematics.
2. I get good grades in mathematics.
3. I learn mathematics quickly.
4. I have always believed that mathematics is one of my best subjects.
5. In my mathematics class, I understand even the most difficult work.

## References

[1] Watkins D.A., Biggs J.B. (2001). Teaching the Chinese Learner: Psychological and Pedagogical Perspectives.

[2] Dello-Iacovo B. (2009). Curriculum reform and ‘quality education’ in China: An overview. Int. J. Educ. Dev..

[3] Alexander R.J. (2001). Culture and Pedagogy: International Comparisons in Primary Education.

[4] Wang D. (2011). The dilemma of time: Student-centered teaching in the rural classroom in China. Teach. Teach. Educ..

[5] Biggs J.B., Watkins D., Biggs J. (1996). Western misperceptions of the Confucian-heritage learning culture. The Chinese Learner: Cultural, Psychological and Contextual Influences.

[6] Sun X., Mainhard T., Wubbels T. (2018). Development and evaluation of a Chinese version of the Questionnaire on Teacher Interaction (QTI). Learn. Environ. Res..

[7] Tan J. (2011). Revisiting the Chinese learner: Changing contexts, changing education. Asia. Pac. J. Educ..

[8] Holm M.E., Björn P.M., Laine A., Korhonen J., Hannula M.S. (2020). Achievement emotions among adolescents receiving special education support in mathematics. Learn. Individ. Differ..

[9] Peixoto F., Sanches C., Mata L., Monteiro V. (2017). “How do you feel about math?”: Relationships between competence and value appraisals, achievement emotions and academic achievement. Eur. J. Psychol. Educ..

[10] Goetz T., Sticca F., Pekrun R., Murayama K., Elliot A.J. (2016). Intraindividual relations between achievement goals and discrete achievement emotions: An experience sampling approach. Learn. Instr..

[11] Pinxten M., Marsh H.W., De Fraine B., Van Den Noortgate W., Van Damme J. (2014). Enjoying mathematics or feeling competent in mathematics? Reciprocal effects on mathematics achievement and perceived math effort expenditure. Brit. J. Educ. Psychol..

[12] Ahmed W., Van der Werf G., Kuyper H., Minnaert A. (2013). Emotions, self-regulated learning, and achievement in mathematics: A growth curve analysis. J. Educ. Psychol..

[13] Goetz T., Frenzel A.C., Pekrun R., Hall N.C., Lüdtke O. (2007). Between-and within-domain relations of students’ academic emotions. J. Educ. Psychol..

[14] Goetz T., Frenzel A.C., Hall N.C., Pekrun R. (2008). Antecedents of academic emotions: Testing the internal/external frame of reference model for academic enjoyment. Contemp. Educ. Psychol..

[15] Luo W., Lee K., Ng P.T., Ong J.X.W. (2014). Incremental beliefs of ability, achievement emotions and learning of Singapore students. Educ. Psychol..

[16] Villavicencio F.T., Bernardo A.B.I. (2013). Positive academic emotions moderate the relationship between self-regulation and academic achievement. Brit. J. Educ. Psychol..

[17] Villavicencio F.T., Bernardo A.B.I. (2016). Beyond math anxiety: Positive emotions predict mathematics achievement, self-regulation, and self-efficacy. Asia-Pacific. Edu. Res..

[18] Dettmers S., Trautwein U., Lüdtke O., Goetz T., Frenzel A.C., Pekrun R. (2011). Students’ emotions during homework in mathematics: Testing a theoretical model of antecedents and achievement outcomes. Contemp. Educ. Psychol..

[19] Frenzel A.C., Thrash T.M., Pekrun R., Goetz T. (2007). Achievement emotions in Germany and China: A cross-cultural validation of the Academic Emotions Questionnaire-Mathematics. J. Cross Cult. Psychol..

[20] Gupta A., Fisher D. (2016). Teacher-student interactions in a technology-supported science classroom environment in relation to selected learner outcomes: An Indian study. MIER J. Educ. Stud. Trends Pract..

[21] Urhahne D. (2015). Teacher behavior as a mediator of the relationship between teacher judgment and students’ motivation and emotion. Teach. Teach. Educ..

[22] Pekrun R. (2006). The control-value theory of achievement emotions: Assumptions, corollaries, and implications for educational research and practice. Educ. Psychol. Rev..

[23] Tellegen A., Clark W.L.A. (1999). On the dimensional and hierarchical structure of affect. Psychol. Sci..

[24] Pekrun R., Perry R.P., Smart J.C. (2007). Emotions in students’ scholastic development. The Scholarship of Teaching and Learning in Higher Education: An Evidence-Based Perspective.

[25] Wang W., Yin H., Lu G., Zhang Q. (2017). Environment matters: Exploring the relationships between the classroom environment and college students’ affect in mathematics learning in China. Asia. Pac. Educ. Rev..

[26] Rimm-Kaufman S.E., Baroody A.E., Larsen R.A.A., Curby T.W., Abry T. (2015). To what extent do teacher-student interaction quality and student gender contribute to fifth graders’ engagement in mathematics learning?. J. Educ. Psychol..

[27] Passini S., Molinari L., Speltini G. (2015). A validation of the Questionnaire on Teacher Interaction in Italian secondary school students: The effect of positive relations on motivation and academic achievement. Soc. Psychol. Educ..

[28] Wubbels T., Brekelmans M., Fraser B.J., Tobin K.G. (1998). The teacher factor in the social climate of the classroom. International Handbook of Science Education.

[29] Misbah Z., Gulikers J., Maulana R., Mulder M. (2015). Teacher interpersonal behaviour and student motivation in competence-based vocational education: Evidence from Indonesia. Teach. Teach. Educ..

[30] Leary T. (1957). Interpersonal Diagnosis of Personality: A Functional Theory and Methodology for Personality Evaluation.

[31] Wubbels T., Brekelmans M., Hooymayers H. (1991). Interpersonal Teacher Behavior in the Classroom.

[32] Krahenbuhl K.S. (2016). Student-centered education and constructivism: Challenges, concerns, and clarity for teachers. Clear. House J. Educ. Strateg. Issues Ideas.

[33] Hallinger P. (2004). Meeting the challenges of cultural leadership: The changing role of principals in Thailand. Discourse-Abingdon.

[34] Kain D.J. (2003). Teacher-centered versus student-centered: Balancing constraint and theory in the composition classroom. Pedagogy.

[35] Dole S., Bloom L., Kowalske K. (2016). Transforming pedagogy: Changing perspectives from teacher-centered to learner-centered. Interdiscip. J. Probl. Based Learn..

[36] Wang H., Zhou X. (2018). Confrontation and Construction of the “Teacher-Centered” and the “Student-Centered”. J. Guizhou Norm. Univ. (Soc. Sci.).

[37] Parsons J., Taylor L. (2011). Improving student engagement. Curr. Issues Educ..

[38] Lee J.C.K., Yin H., Zhang Z. (2009). Exploring the influence of the classroom environment on students’ motivation and self-regulated learning in Hong Kong. Asia-Pac. Educ. Res..

[39] Perry R.P., Hladkyj S., Pekrun R.H., Pelletier S.T. (2001). Academic control and action control in the achievement of college students: A longitudinal field study. J. Educ. Psychol..

[40] Maulana R., Opdenakker M.C., den Brok P., Bosker R. (2011). Teacher-student interpersonal relationships in Indonesia: Profiles and importance to student motivation. Asia. Pac. J. Educ..

[41] Telli S., den Brok P., Cakiroglu J. (2008). Teacher-Student Interpersonal Behavior in Secondary Science Classes in Turkey. J. Classr. Interact..

[42] Goetz T., Pekrun R., Hall N., Haag L. (2006). Academic emotions from a social-cognitive perspective: Antecedents and domain specificity of students’ affect in the context of Latin instruction. Brit. J. Educ. Psychol..

[43] Mitchell M.M., Bradshaw C.P., Leaf P.J. (2010). Student and teacher perceptions of school climate: A multilevel exploration of patterns of discrepancy. J. Sch. Health.

[44] Tshewang R., Chandra V., Yeh A. (2017). Students’ and teachers’ perceptions of classroom learning environment in Bhutanese eighth-grade mathematics classes. Learning. Environ. Res..

[45] Fisher D., Fraser B.J., Wubbels T., Wubbels T., Levy J. (1993). Interpersonal teacher behavior and school environment. Do You Know What You Look Like: Interpersonal Relationships in Education.

[46] Podsakoff P.M., MacKenzie S.C., Lee J.-Y., Podsakoff N.P. (2003). Common method biases in behavioral research: A critical review of the literature and recommended remedies. J. Appl. Psychol..

[47] Schreiber J.B., Nora A., Stage F.K., Barlow E.A., King J. (2006). Reporting structural equation modeling and confirmatory factor analysis results: A review. J. Educ. Res..

[48] Hayes A.F. (2009). Beyond Baron and Kenny: Statistical mediation analysis in the new millennium. Commun. Monogr..

[49] Edwards J.R., Caplan R.D., Harrison R.V., Cooper C.L. (1998). Person-environment fit theory: Conceptual foundations, empirical evidence, and directions for future research. Theories of Organizational Stress.

[50] Lee J.C.K., Yin H. (2010). The impact of classroom environment on students’ self-regulated learning in Hong Kong: A commentary on the debate of teacher-centeredness and student-centeredness. Peking Univ. Educ. Rev..

[51] Mok I.A.C., Chik P.P.M., Ko P.Y., Kwan T.Y.L., Lo M.L., Marton F., Ng D.F.P., Pang M.F., Runesson U., Sze-To L.H., Watkins D.A., Biggs J.B. (2001). Solving the paradox of the Chinese teacher. Teaching the Chinese Learner: Psychological and Pedagogical Perspectives.

[52] Gao L., Watkins D.A., Watkins D.A., Biggs J.B. (2007). Towards a model of teaching conceptions of Chinese secondary school teachers of physics. Teaching the Chinese Learner: Psychological and Pedagogical Perspectives..

[53] Meinhardt J., Pekrun R. (2003). Attentional resource allocation to emotional events: An ERP study. Cogn. Emot..

[54] Pekrun R., Goetz T., Titz W., Perry R.P. (2002). Academic emotions in students’ self-regulated learning and achievement: A program of qualitative and quantitative research. Educ. Psychol..

[55] Goetz T., Hall N.C., Hattie J., Anderman E.M. (2013). Emotion and achievement in the classroom. International Guide to Student Achievement.

[56] Pekrun R., Linnenbrink-Garcia L. (2014). Introduction to emotions in education. International Handbook of Emotions in Education.

[57] Wu H.K., Huang Y.L. (2007). Ninth-grade student engagement in teacher-centered and student-centered technology-enhanced learning environments. Sci. Educ..

